# Process analysis and optimization of simultaneous saccharification and co-fermentation of ethylenediamine-pretreated corn stover for ethanol production

**DOI:** 10.1186/s13068-018-1118-8

**Published:** 2018-04-23

**Authors:** Lei Qin, Xiong Zhao, Wen-Chao Li, Jia-Qing Zhu, Li Liu, Bing-Zhi Li, Ying-Jin Yuan

**Affiliations:** 1 Key Laboratory of Systems Bioengineering (Ministry of Education), School of Chemical Engineering and Technology, Tianjin University, Weijin Road 92, Nankai District, Tianjin, 300072 People’s Republic of China; 2 SynBio Research Platform, Collaborative Innovation Center of Chemical Science and Engineering (Tianjin), Tianjin University, Weijin Road 92, Nankai District, Tianjin, 300072 People’s Republic of China; 3 Present Address: Institute for Synthetic Biosystem/Department of Biochemical Engineering, School of Chemistry and Chemical Engineering, Beijing Institute of Technology, ZhongGuanCunNan Road 5, Beijing, People’s Republic of China

**Keywords:** Biomass, Ethylenediamine pretreatment, Cellulosic ethanol, Xylose fermentation, *Saccharomyces cerevisiae*, Enzyme recycle

## Abstract

**Background:**

Improving ethanol concentration and reducing enzyme dosage are main challenges in bioethanol refinery from lignocellulosic biomass. Ethylenediamine (EDA) pretreatment is a novel method to improve enzymatic digestibility of lignocellulose. In this study, simultaneous saccharification and co-fermentation (SSCF) process using EDA-pretreated corn stover was analyzed and optimized to verify the constraint factors on ethanol production.

**Results:**

Highest ethanol concentration was achieved with the following optimized SSCF conditions at 6% glucan loading: 12-h pre-hydrolysis, 34 °C, pH 5.4, and inoculum size of 5 g dry cell/L. As glucan loading increased from 6 to 9%, ethanol concentration increased from 33.8 to 48.0 g/L, while ethanol yield reduced by 7%. Mass balance of SSCF showed that the reduction of ethanol yield with the increasing solid loading was mainly due to the decrease of glucan enzymatic conversion and xylose metabolism of the strain. Tween 20 and BSA increased ethanol concentration through enhancing enzymatic efficiency. The solid-recycled SSCF process reduced enzyme dosage by 40% (from 20 to 12 mg protein/g glucan) to achieve the similar ethanol concentration (~ 40 g/L) comparing to conventional SSCF.

**Conclusions:**

Here, we established an efficient SSCF procedure using EDA-pretreated biomass. Glucose enzymatic yield and yeast viability were regarded as the key factors affecting ethanol production at high solid loading. The extensive analysis of SSCF would be constructive to overcome the bottlenecks and improve ethanol production in cellulosic ethanol refinery.

## Background

To meet the global demand for energy, lignocellulosic biomass has long been recognized as a potential sustainable resource of mixed sugars for bioethanol fermentation [1]. Corn stover (CS) is an abundant agricultural residue, which makes it a suitable feedstock for cellulosic ethanol production. The biorefinery process of CS to ethanol was composed of pretreatment, enzymatic hydrolysis, fermentation, and purification. In the light of the commercial viability, it is necessary to optimize the biorefinery process, reduce cost, and increase ethanol concentration and productivity, which are bottlenecks in bioethanol refinery [2].

The choice of pretreatment has a significant impact on biorefinery costs and on most other downstream processing decisions [3]. Ethylenediamine (EDA) pretreatment preserves most of sugars in biomass and provides high enzymatic digestibility. It can be operated at ambient pressures and high solid-to-liquid ratios to realize a “dry-to-dry” process [4, 5]. EDA has just the appropriate boiling point (117 °C) to react with biomass and separate from biomass simultaneously. After EDA pretreatment, most of cellulose and hemicellulose remained in water insoluble solid fraction. Lignin is relocated to the surface of biomass with ether and ester bonds in lignin–hemicellulose complex cleaved, and partial crystal cellulose is transformed to amorphous cellulose [4]. Glucose enzymatic yield of pretreated CS can be over 90% [5]. Washing prior to enzymatic hydrolysis, a detoxification process, is used to further improve the enzymatic hydrolysis and reduce enzyme dosage, since enzymatic hydrolysis is inhibited by soluble oligosaccharides and phenolics derived from pretreated CS [6, 7].

Simultaneous saccharification and co-fermentation (SSCF) is a promising process for bioethanol refinery, in which enzymatic hydrolysis of the pretreated biomass occurs simultaneously with the co-fermentation of hexose and pentose (mainly glucose and xylose) by genetically engineered *Saccharomyces cerevisiae*, as it enables reducing investment cost, saving energy, and achieving higher ethanol productivity by reducing end-products inhibition compared with separate hydrolysis and co-fermentation (SHCF) [8–10]. As the conditions to achieve highest ethanol concentration may be varied when using different lignocellulosic substrates, pretreatments, or microbes, it is imperative to investigate optimal conditions of SSCF with EDA-pretreated CS, [11–15]. In addition, high solid loading is prerequisite for achieving high ethanol concentration, which is beneficial to reduce the cost of distillation. However, ethanol yield decreased as solid loading increased probably due to the lack of enzyme activity, the deficiency of cell viability, or other unknown factors [10, 16, 17]. Mass balance data of SSCF would be useful to find reasons for the decrease of ethanol yield and seek out corresponding tactics.

In this study, we comprehensively analyzed the critical factors and optimized ethanol concentration in SSCF with EDA-pretreated CS for the first time. We found that the glucose enzymatic yield and the xylose utilization of the strain were seriously affected by solid loading. Furthermore, in view of high enzyme dosage in traditional SSCF process, we carried out an enzyme-recycled SSCF strategy to reduce enzyme dosage and production period. Recycling solid residues in SSCF is an advisable recycling strategy to recycle unhydrolyzed carbohydrates, adsorbed enzymes, and yeast cells. It was reported that alkaline-pretreated biomass was more suitable for enzyme recycling than acid-pretreated biomass probably due to the lower lignin content in alkaline-pretreated biomass [18]. By the recycling strategy, enzyme dosage can be decreased by about 30% as reported previously [19–21]. The optimized SSCF with EDA-pretreated biomass from this report would be referable and constructive to improve efficiency of cellulosic ethanol refinery.

## Methods

### Materials

Corn stover (CS), harvested in the suburb of Tianjin, China, was air-dried and milled. Particle size between 0.2  and   2 mm was sieved and collected for pretreatment. The moisture of the prepared CS was 4–6%. EDA (≥ 99%) was purchased from Yuanli Co. (Tianjin, CN). Commercial enzymes Cellic Ctec2 and Cellic Htec2 were gifted by Novozymes (Beijing, CN).

### Ethylenediamine pretreatment

EDA pretreatment was carried out in an electric oven as previously reported [5]. In brief, CS was filled into a 1-L beaker and mixed with pure EDA (0.8 mL/g CS). The beaker was put into the oven (with a fume hood at the air outlet) at 130 °C. After 20-min residence time, the mixture was poured on a stainless-steel tray and manually stirred every 5 min at 130 °C for 40 min. Biomass was then cooled down at room temperature and underwent a minor washing step (washed twice with 10 mL distilled water per g solid). The final pretreated CS was dried at room temperature until the moisture was less than 10%.

### Microorganism and seed culture preparation

Engineered xylose-fermenting strain *S. cerevisiae* SyBE005 was constructed and further mutated by evolutionary engineering in our previous research [22]. Seed culture was prepared by inoculating a single colony of genetic yeast from a YPX-Agar plate (per liter: 10 g yeast extract, 20 g peptone, 20 g xylose, and 20 g agar) into a tube containing 5 mL YPX medium (per liter: 10 g yeast extract, 20 g peptone and 20 g xylose) at 30 °C, 250 rpm for 24 h; and then 500 µL culture was inoculated into 100 mL YPX medium in a 250-mL flask and incubated for 18 h at 30 °C and 250 rpm. The final OD_600_ was around 5 (equal to 2.5 g dry cell/L). The relationship between cell turbidity and dry cell weight was determined according to previous literature [23]. The cells were harvested for fermentation inoculation by centrifugation at 1500×*g* for 5 min.

### Simultaneous saccharification and co-fermentation (SSCF)

SSCF were performed in 100-mL flask with 20 g total mixture at 150 rpm. The biomass was first pre-hydrolyzed using the commercial enzymes with designated pH (4.4, 4.8, or 5.4) and time (0, 12, or 24 h) at 50 °C and 250 rpm. pH was adjusted by concentrated sulfuric acid or potassium hydroxide. Cellic Ctec2 and Cellic Htec2 were added with a loading of both 10 mg protein/g glucan. Ampicillin with a final concentration of 50 mg/L was used to prevent bacterial contamination. After the pre-hydrolysis, yeast cells were inoculated at designated inoculum sizes (0.5, 2.5, or 5 g dry cell/L). The temperature was adjusted to designated values (30, 34, or 38 °C). Shaker speed was 150 rpm. Syringe needles were pierced through the rubber plug to release carbon dioxide during the first 12-h SSCF. 0.5 mL samples were withdrawn at 0, 12, 24, 48, and 72 h during SSCF to determine glucose, xylose, and ethanol concentration.

For SSCF with 6 and 9% glucan loadings, non-ionic surfactant Tween 20 or bovine serum albumin (BSA) was added at the beginning of pre-hydrolysis with a concentration of 0.1 g/L.

### Analytical methods

The compositions of pretreated and raw CS were determined following the laboratory analytical procedure (LAP) of the National Renewable Energy Laboratory. Compositions of untreated and pretreated CS are summarized in Table 1. Glucose, xylose, arabinose, and ethanol concentrations were analyzed using HPLC equipped with a refractive index detector (Waters 410) and a Bio-Rad Aminex HPX-87H column. The column temperature was 65 °C. Mobile phase (5 mM H_2_SO_4_) flow rate was 0.6 mL/min. Mannose and galactose, co-eluted with xylose peak, were ignored in this study, as the contents of mannan and galactan in pretreated CS were lower than 2% which were determined by an Aminex HPX-87P column. Gluco- and xylo-oligomer concentrations were also determined according to LAP method. In brief, enzymatic hydrolysates containing oligomers were completely hydrolyzed with 4% H_2_SO_4_ at 120 °C for 1 h, and the hydrolysis products (glucose and xylose) were determined by HPLC.

**Table 1:** Compositions of CS

	Untreated CS	EDA-pretreated CS
Glucan	39.6% ± 0.7%	45.2% ± 1.0%
Xylan	23.2% ± 0.3%	21.7% ± 0.5%
Arabinan	3.5% ± 0.2%	7.4% ± 0.3%
Acid-insoluble lignin	17.7% ± 0.4%	8.1% ± 0.3%
Acid-soluble lignin	0.7% ± 0.1%	7.6% ± 0.2%
Acetyl	2.6% ± 0.3%	0.0% ± 0.0%
Ash	7.2% ± 0.3%	7.8% ± 0.4%
Protein	5.4% ± 0.4%	2.5% ± 0.3%
Lipid	4.0% ± 0.3%	2.0% ± 0.2%
Total	103.9%	102.3%

The protein concentration of enzymes was determined by the BCA protein assay. Cellulase activity was determined using Whatman No.1 filter paper as described by Ghosh [24]. Xylanase activity was determined using xylan (reagent grade, 90%, Sigma-Aldrich, US) according to Bailey et al. [25]. Enzyme concentrations and activities are shown in Table 2.

**Table 2:** Enzyme concentrations and activities

Enzymes	Cellic Ctec2	Cellic Htec2
Protein concentration (mg/mL)	182	198
Cellulase activity (FPU/mL)^a^	77	11
Xylanase activity (IU/mL)^b^	160	81

^a^FPU stands for filter paper units. One FPU of enzyme is defined as the amount of enzyme catalyzing the release of 1 μmol of glucose equivalent per min

^b^One IU of xylanase is defined as the amount of enzyme catalyzing the release of 1 μmol of xylose equivalent per min

### Calculations

To facilitate the mass balance calculation, contents of glucan and xylan in the pretreated CS and solid residue were converted into the content of glucose and xylose. Ethanol yield was calculated based on total sugars in pretreated CS, and ethanol metabolic yield was based on available sugars in hydrolysate. Glucan conversion, xylan conversion, ethanol yield, and ethanol metabolic yield are calculated as follows:

1 $${\text{Glucan}}/{\text{xylo}}\;{\text{conversion }} (\%) = \left[ {{\text{Glucose}}/{\text{xylose}}\;{\text{in}}\;{\text{presented}}\;{\text{CS }}({\text{g}}) -\, {\text{gluco/xylo}}-{\text{oligomer }}({\text{g}})-{\text{glucose}}/{\text{xylose}}\;{\text{in}}\;{\text{residue }}({\text{g}})} \right]/{\text{glucose}}/{\text{xylose}}\;{\text{in}}\;{\text{presented}}\;{\text{CS }}({\text{g}}) \times 100\%$$

2 $${\text{Ethanol}}\;{\text{yield}} (\% ) = {\text{Ethanol}}\;{\text{in}}\;{\text{broth }}({\text{g}})/\left[ {0.51 \times {\text{glucose}}\;{\text{and}}\;{\text{xylose}}\;{\text{in}}\;{\text{presented}}\;{\text{CS }}({\text{g}})} \right] \times 100\%$$

3 $${\text{Ethanol}}\;{\text{metabolic}}\;{\text{yield }}(\%) = {\text{Ethanol}}\;{\text{in}}\;{\text{broth }}({\text{g}})/\left\{ {0.51 \times \left[ {{\text{glucose}}\;{\text{and}}\;{\text{xylose}}\;{\text{in}}\;{\text{presented}}\;{\text{CS }}({\text{g}}) - {\text{glucose}}\;{\text{and}}\;{\text{xylose}}\;{\text{in}}\;{\text{solid}}\;{\text{residue}}\;({\text{g}})-{\text{glucose}}\;{\text{and}}\;{\text{xylose}}\;({\text{oligomers}})\;{\text{in}}\;{\text{broth}}\,({\text{g}})} \right]} \right\} \times 100\%$$

### Recycling SSCF process

SSCF with the recycling of solid residue was carried out as shown in Fig. 4. Pre-hydrolysis was conducted with 9% glucan loading, 50 °C, and 250 rpm for 24 h (for the purpose of quickly obtaining 40 g/L ethanol and sufficient liquefaction). Enzymes were added at one time. Ctec2 and Htec2 loading were both 10 mg protein/g glucan. Pretreated CS was added in three portions (3.8, 2.6, and 2.6% glucan loading at 0, 6, and 12 h, respectively). After pre-hydrolysis, yeast cell was inoculated at 5 g dry cell/L. SSCF condition was pH 5.5, 34 °C, and 150 rpm. After 48 h, SSCF broth was centrifuged at 2000×*g* for 20 min. The supernatant was frozen at − 20 °C for component analysis. The residual solid was inoculated into the next cycle flask of pre-hydrolysate and thereby the total volume increased from cycle to cycle. 0, 30, 50, 70, and 100% of enzyme loading of cycle 1 was added into the beginning of pre-hydrolysate of cycle 2 to find the proper enzyme dosage to acquire comparable ethanol concentration with cycle 1. This fresh enzyme dosage was used for subsequent cycles (2–5). After five cycles, water was added into the residue solids without enzymes and fresh biomass supplementary for the last step.

## Results

### Optimal conditions of SSCF

**Fig. 1:**
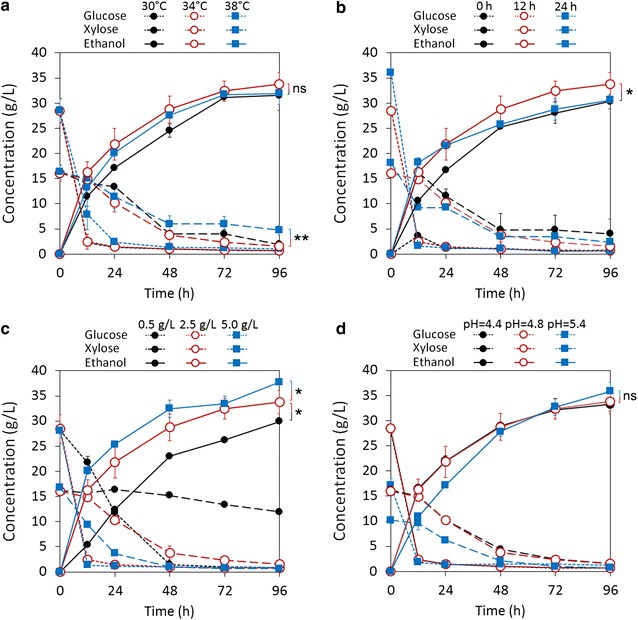
Effect of temperature (**a**), pre-hydrolysis time (**b**), inoculum size (**c**), and pH (**d**) on SSCF of EDA-pretreated CS. The default conditions were 6% glucan loading, temperature 34 °C, pH = 4.8, inoculum size 2.5 g dry cell/L, 12-h pre-hydrolysis, and 150 rpm shaker speed. Ctec2 and Htec2 loadings were both 10 mg protein/g glucan. Error bars represent SD, *n *= 3, ns: not significant, *P < 0.05, **P < 0.01

SSCF of EDA-pretreated CS was carried out with an engineered xylose-fermenting strain *S. cerevisiae* SyBE005. To identify the key factors and maximize the ethanol concentration, several factors in SSCF were investigated (including temperature, pre-hydrolysis time, pH, and inoculum size) (Fig. 1). A pre-hydrolysis process was performed followed by SSCF to provide a basic carbon source for yeast and better mass transfer at 0 h of SSCF.

SSCF at 34 °C had a slightly higher ethanol concentration (33.7 g/L) at 96 h than that of 30 °C (31.6 g/L) and 38 °C (31.9 g/L). Compared to 38 °C, 30 and 34 °C induced to significant higher xylose consumptions (Fig. 1a). Ethanol concentration at 96 h reached the maximum with 12-h pre-hydrolysis, although higher ethanol concentration was achieved with 24-h pre-hydrolysis within initial 12-h SSCF (Fig. 1b). As inoculum size increasing from 0.5 to 5 g dry cell/L, ethanol concentration (96 h) increased from 29.9 to 37.8 g/L, and xylose concentration (96 h) decreased from 11.9 to 0.6 g/L (Fig. 1c). Ethanol concentration (96 h) reached 35.9 g/L at pH 5.4, which was slightly higher than that of pH 4.4 and 4.8, in spite of glucose and xylose concentrations at 0 h of pH 5.4 were much lower than that of pH 4.4 and 4.8 (Fig. 1d).

### Effect of glucan loading on SSCF

**Fig. 2:**
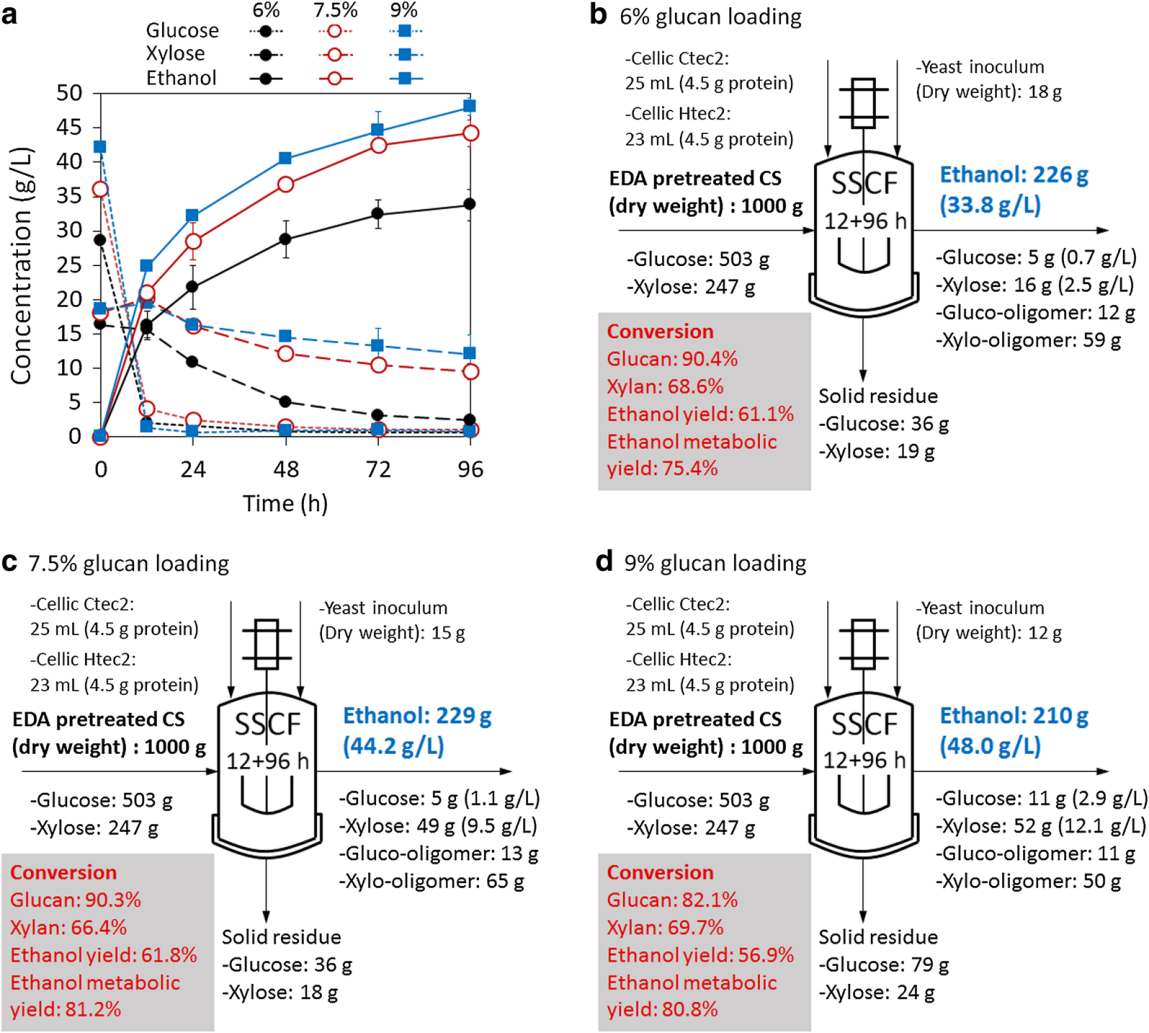
Effect of glucan loading (6, 7.5, and 9%) on SSCF of EDA-pretreated CS. **a** Glucose, xylose, and ethanol concentrations during SSCF. Error bars represent SD, *n *= 3. **b**–**d** Mass balance of SSCF with 6, 7.5, and 9% glucan loading. Other conditions were temperature 34 °C, pH = 4.8, inoculum size 2.5 g dry cell/L, 12-h pre-hydrolysis, and 150 rpm shaker speed. Ctec2 and Htec2 loadings were both 10 mg protein/g glucan

To improve ethanol concentration, increasing solid loading was essential in SSCF. Ethanol concentrations with different glucan loadings (6, 7.5, and 9%) at 96 h reached 33.8, 44.2, and 48.0 g/L, respectively. Higher xylose concentrations (96 h) remained for 7.5 and 9% glucan loadings (9.5 and 12.1 g/L) when compared to 6% glucan loading (2.5 g/L) (Fig. 2a). Mass balance was conducted for each glucan loading to evaluate sugar conversions and ethanol yields (Fig. 2b–d). Both glucan conversion and ethanol yield at 9% glucan loading were decreased when compared to 6 and 7.5% glucan loading. No significant change of xylan conversion was observed. However, ethanol metabolic yield (calculated based on consumed glucose and xylose) was higher at 7.5 and 9% glucan loading (81.2 and 80.8%, respectively) than 6% glucan loading (75.4%). This result suggests that increasing solid loading inhibits enzymatic hydrolysis of glucan but not xylan. Increasing solid loading also inhibits xylose metabolism but stimulates metabolic flux of ethanol fermentation. 1 kg pretreated CS produced 226, 229, and 210 g ethanol for 6, 7.5, and 9% glucan loading, respectively. As a result, SSCF with 9% glucan loading exhibited 42% improvement in ethanol concentration and 7% reduction of ethanol yield compared with 6% glucan loading.

### Effect of additives on SSCF

**Fig. 3:**
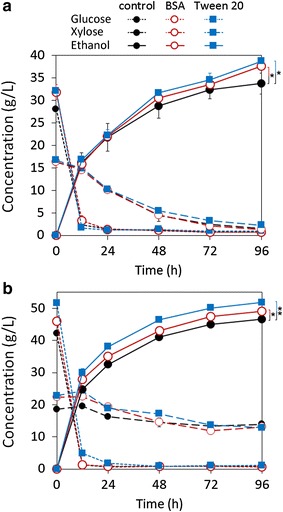
Effect of BSA and Tween 20 addition on SSCF of EDA-pretreated CS. **a** 6% glucan loading; **b** 9% glucan loading. SSCF conditions were temperature 34 °C, pH = 4.8, inoculum size 2.5 g dry cell/L, 12-h pre-hydrolysis, and 150 rpm shaker speed. Ctec2 and Htec2 loadings were both 10 mg protein/g glucan. The concentration of BSA and Tween 20 was 0.1 g/L. Error bars represent SD, *n *= 3, *P < 0.05, **P < 0.01

The effects of Tween 20 and BSA on SSCF were investigated (Fig. 3). With the addition of Tween 20 and BSA, glucose concentrations at the end of pre-hydrolysis increased by 13.5 and 14.6% at 6% glucan loading and increased by 9.1 and 22.4% at 9% glucan loading, respectively. Ethanol concentrations at 96 h with the addition of Tween 20 and BSA were consequently increased (14.7 and 11.5% higher than the control at 6% glucan loading, and 5.5 and 11.2% higher than the control at 9% glucan loading, respectively). Whereas, glucose and xylose consumption did not boost with the addition of Tween 20 or BSA, implying that the additives had no obvious influence on yeast metabolism. Tween 20 and BSA have been reported to promote cellulose hydrolysis through enhancing cellulase stability and specific adsorption of cellulase to cellulose, especially in the presence of lignin [26, 27]. As a result, the improved ethanol concentrations were due to the increased sugar yields of enzymatic hydrolysis.

### Recycling SSCF process

**Fig. 4:**
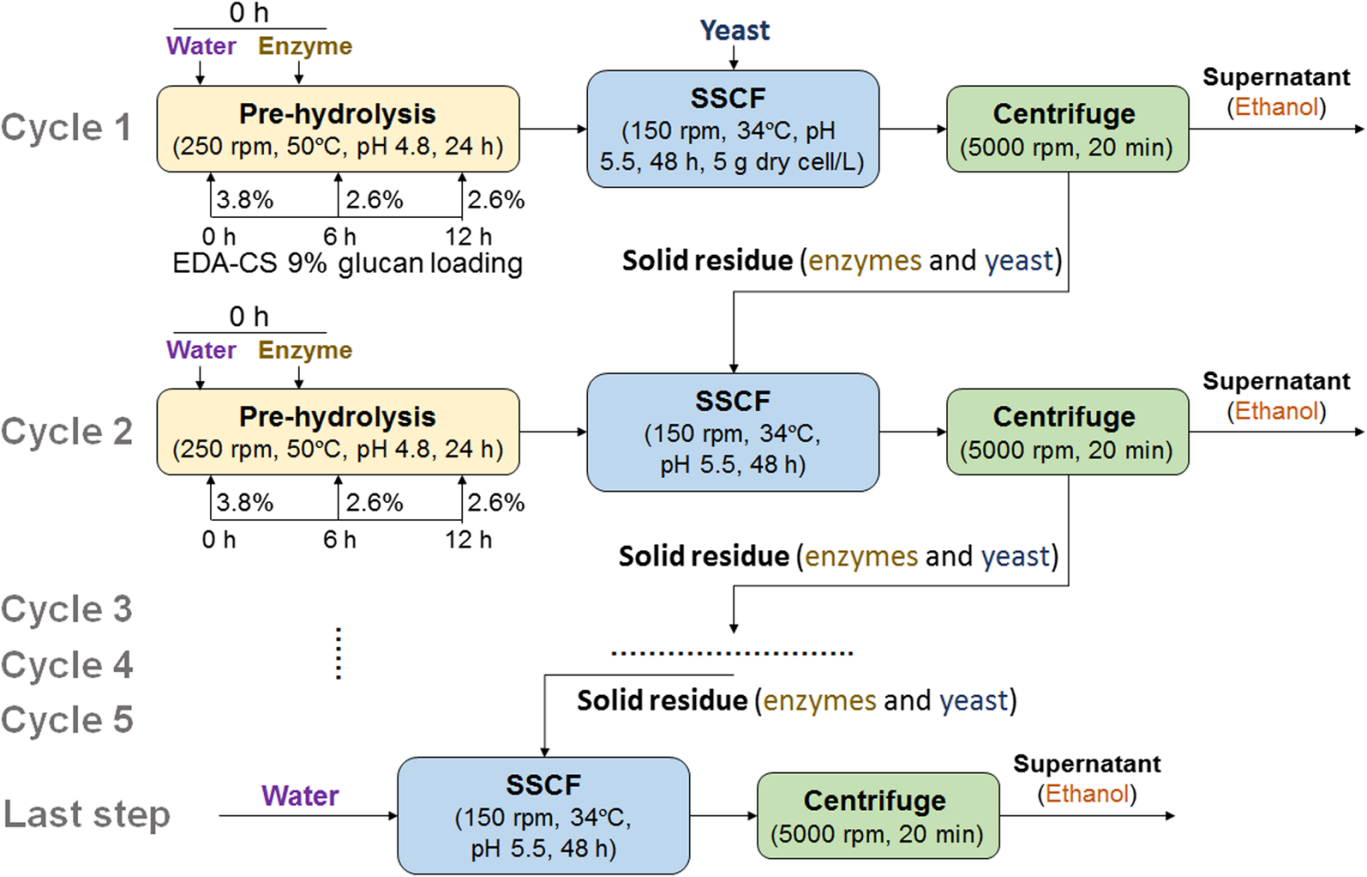
Flow diagram of recycling SSCF process of pretreated CS

To reduce enzyme dosage and fermentation time, enzyme-recycled SSCF strategy was adopted as shown in Fig. 4. We carried out 24-h pre-hydrolysis with 9% glucan loading of each cycle followed by 48-h SSCF to obtain ~ 40 g/L ethanol in a relatively short time. Pretreated CS was added in batches within 12 h. Residual solid after SSCF was centrifuged and transferred to the next cycle’s SSCF. In pre-hydrolysis of cycle 2–5, enzyme loadings in pre-hydrolysis were reduced to save enzyme as another part of enzyme activity came from the adsorbed enzyme in the solid residue of previous cycle. Solid residue also offered unhydrolyzed carbohydrates for further hydrolysis and seed cells for fermentation. Reaction volumes and solid concentrations thereby increased cycle by cycle.

**Fig. 5:**
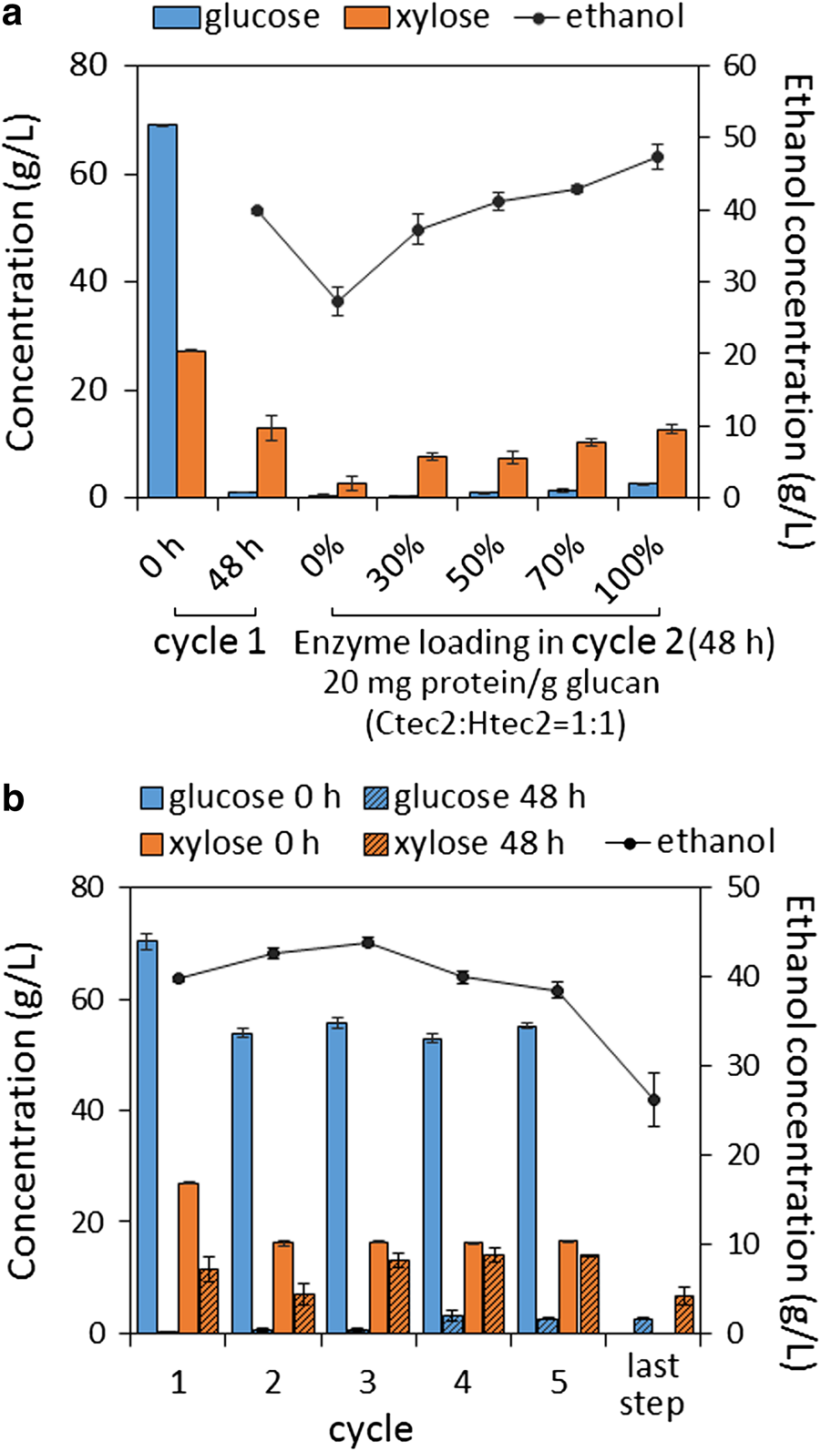
Performance of recycling SSCF process. **a** Determination of enzyme loading for cycle 2. Glucose, xylose, and ethanol concentrations after 48-h SSCF of cycle 2 with different enzyme loadings (0, 30, 50, 70, and 100%). Enzyme loading for cycle 1 is 20 mg/g glucan (Ctec2: Htec2 = 1:1) (100%). **b** Glucose, xylose, and ethanol concentrations before and after SSCF of each cycle. Enzyme loadings for cycles 2–5 were 50% of the cycle 1 and no enzyme loadings for the last step. Error bars represent SD, *n *= 2

Experiments were performed to optimize the enzyme loading (0, 30, 50, 70, and 100% of cycle 1) in cycle 2 to obtain similar ethanol concentration to cycle 1 (39.8 g/L at 48 h) (Fig. 5a). Without adding fresh enzymes (0%), 27.3 g/L ethanol was achieved after 48-h SSCF of cycle 2, suggesting enzymes in solid residue worked. As fresh enzyme loading increased in pre-hydrolysis of cycle 2, ethanol concentration at 48-h SSCF increased. When 50% fresh enzyme of the cycle 1 was added into cycle 2, similar ethanol concentration (41.1 g/L) was obtained. Therefore, this enzyme loading (10 mg total protein/g glucan) was used for cycle 2–5, resulting in a 50% enzyme saving for cycle 2–5.

In each of the cycles (2–5), pre-hydrolysis produced similar glucose and xylose concentrations (0 h of SSCF) (Fig. 5b). After each cycle, most of glucose was consumed but residual xylose increased along with cycles. Ethanol concentration increased to a maximum at cycle 3 (43.9 g/L) and began to drop from cycle 3 to cycle 5 (38.5 g/L in cycle 5), indicating that the recycling process was unable to maintain for more cycles. The concentration of residual xylose (48 h) increased from 7.0 g/L (cycle 2) to above 13.0 g/L (cycle 3–5), which might be a reason for the decline of ethanol concentrations. The last step of recycling SSCF was performed without solids and fresh enzyme addition, which was hard to produce high ethanol concentration.

## Discussion

In this work, SSCF conditions of EDA-pretreated CS were optimized, which produced the highest ethanol concentration at 34 °C, 12-h pre-hydrolysis, pH 5.4, and inoculum size of 5 g dry cell/L. We found that the optimum conditions of SSCF might be quite different among various cases due to the different compositions of pretreated biomass, amounts of by-products, and microbial robustness. As known, *S. cerevisiae* grows at about 30 °C, while carbohydrate-active enzymes have optimum temperatures of around 50 °C. High temperature leads to increased mortality of yeast, while low temperature leads to reduced enzymatic hydrolysis rate. Our previous study showed that the highest ethanol productivity was achieved at 38 °C using aqueous ammonia-pretreated CS by *S. cerevisiae* SyBE005 [12]. Synergistic effect of high temperature and inhibitors in dry dilute acid-pretreated CS reduced the xylose consumption rate and cell viability of SyBE005 and reduced the optimum temperature to 34 °C [13]. The result of this study also supports that xylose consumption is more susceptible by increased temperature (Fig. 1a). Rudolf et al. [28] indicated that more xylose was consumed by *S. cerevisiae* TMB3400 at 32 °C in comparison with 37 °C. Whereas, other researchers showed that the highest ethanol yields were achieved at over 37 °C using thermal-tolerant yeasts [11, 29]. It was indicated that longer pre-hydrolysis time induced higher glucose concentration and thus higher cell viability and xylose consumption rate using *S. cerevisiae* 424A(LNH-ST) [30]. However, other researches, including our result (Fig. 1b), showed that longer pre-hydrolysis time resulted in a larger decrease in overall ethanol yield than shorter pre-hydrolysis time. It was considered that enzyme deactivation in pre-hydrolysis and osmotic stress response to high glucose concentration were responsible for this phenomenon [12, 31]. A previous study showed that higher ethanol concentration and xylose consumption were achieved at high pH value (5.5) [14], which agrees with the finding in this work (Fig. 1d). The result implies that pH adjustment in SSCF is needed. For example, pH value is 4.8 in pre-hydrolysis and adjusted to 5.5 after inoculation. Our result showed that higher inoculum size permitted higher xylose consumption rate and ethanol concentration (Fig. 1c). This agrees with a previous study, in which highest ethanol concentration was achieved at inoculation size of about 8 g dry cell/L [14].

Improving ethanol concentration is the permanent goal of bioethanol refinery. Ethanol concentration should be as high as possible to reduce distillation cost, although 4% (v/v, equivalent to 32 g/L) ethanol is the threshold allowed to be distilled. Therefore, SSCF with high solid loading is necessary and becomes a bottleneck in cellulosic ethanol, as several studies found that ethanol yield decreased with increasing solid loading [10, 12, 16, 29]. In this study, ethanol yield of SSCF of EDA-pretreated CS also showed a slight reduction when glucan loading increased from 6 to 9%. We identified that it was mainly due to the significant decrease of glucan enzymatic conversion and xylose metabolism, although ethanol metabolic yield slightly increased with increasing glucan loading (Fig. 2). This result agrees with our previous results about the addition of water extract (mainly contains soluble lignin and xylo-oligomer) on SSCF, in which we found that these soluble materials from pretreated biomass inhibited enzymatic hydrolysis but stimulated fermentation, and this dual effect of solubles led to a decrease of ethanol yield at high solid loadings [32]. Through adding Tween 20 or BSA, ethanol concentration effectively increased by 5–15% (Fig. 3), which suggests that enzymatic hydrolysis is a rate-limiting step in high solid loading SSCF. Another reason for decreased ethanol yield is the reduced xylose consumption (Fig. 2), which was probably caused by (1) increased concentration of soluble inhibitors, such as lignocellulose degradation products (phenolics), fermentation metabolites, and ethanol [30, 32]; (2) reduced ratio of inoculum size to solid concentration. We kept inoculum size constant among different glucan loadings, while the ratio of inoculum size to glucan loading decreased when glucan loading increases. In this situation, xylose consumption obviously decreased, so we considered that this ratio might play an important role in cell viability and xylose consumption. Therefore, increasing enzymatic hydrolysis efficiency and cell viability will be the key points for cellulosic ethanol production.

As the cost of cellulase accounts for a considerable proportion in the whole cost of bioethanol refinery, reducing enzyme dosage is another bottleneck. Although surfactants or BSA can significantly improve ethanol yield and reduce enzyme dosage, it is still very costly in industry. To reduce enzyme dosage instead, enzyme-recycled process seems to be highly desirable. By adding the solid residue from SSCF into the next SSCF unit without fresh enzyme addition, we got 27.3 g/L ethanol (Fig. 5a), suggesting that there was still a part of enzyme of good activity in residues. Therefore, solid residues can be recycled in the following cycles with supplementation of only 50% fresh enzyme of cycle 1. The average enzyme loading of the recycling SSCF with five cycles was 12 mg protein/g glucan (equivalent to 5.4 mg protein/g solid), which decreased enzyme dosage by 40% to achieve the similar ethanol concentration with the conventional SSCF process. This enzyme loading was much lower than that in previous studies about recycling SSCF using AFEX- and dilute acid-pretreated biomass [19, 21], exhibiting excellent digestibility and fermentability of EDA-pretreated biomass. It is worth noting that yeast cells were also recycled, which saves a part of cost of seed culture. The 5-cycle SSCF showed that ethanol concentration began to drop from cycle 3. There may be some conditions deteriorated with the recycles that influenced SSCF efficiency: (1) increased solid concentration and expanded reaction volume resulted in reduced mass transfer [19]; (2) increased concentrations of soluble materials and carbohydrates inhibited enzyme activity and cell viability [33]. It was found that xylose consumption decreased cycle by cycle (from 15.4 g/L at cycle 1 to 2.5 g/L at cycle 5, Fig. 5b), indicating that cell viability decreased and became the limiting factor in SSCF. To further extend cycle numbers, fresh seed cells need to be added to maintain the cell viability and xylose consumption rate. Even so, the recycling SSCF process exhibited a 40% reduction of enzyme cost compared with conventional SSCF process.

## Conclusions

We established a SSCF process with EDA-pretreated CS and identified the optimum conditions. The optimization of SSCF effectively improved ethanol concentration. Ethanol concentration of 9% glucan loading SSCF demonstrated 42% increase of ethanol concentration and 7% decrease of ethanol yield compared with 6% glucan loading. We analyzed that the decrease of ethanol yield with increasing glucan loading was mainly due to the declined enzymatic efficiency for glucan and the declined xylose consumption. Recycling SSCF process with five cycles reduced enzyme dosage from 20 to 12 mg protein/g glucan, which saves 40% enzyme cost accordingly. This study demonstrated the high efficiency of EDA pretreatment coupled with SSCF process in biorefinery of cellulosic ethanol.

## Abbreviations

SSCF: simultaneous saccharification and co-fermentation
EDA: ethylenediamine
CS: corn stover
BSA: bovine serum albumin
LAP: laboratory analytical procedure
FPU: filter paper unit
